# DSPE-PEG Modification of α-Conotoxin TxID

**DOI:** 10.3390/md17060342

**Published:** 2019-06-08

**Authors:** Weinan Zhao, Yang Xiong, Dongting Zhangsun, Sulan Luo

**Affiliations:** Key Laboratory of Tropical Biological Resources, Ministry of Education, Key Laboratory for Marine Drugs of Haikou, Hainan University, Haikou 570228, China; zzhaoeee@163.com (W.Z.); ncdxxy@163.com (Y.X.); zhangsundt@163.com (D.Z.)

**Keywords:** α-conotoxin TxID, PEG modification, stability, α3β4 nAChR

## Abstract

In order to improve stability of a peptide marine drug lead, α-conotoxin TxID, we synthesized and modified TxID at the N-terminal with DSPE-PEG-NHS by a nucleophilic substitution reaction to prepare the DSPE-PEG-TxID for the first time. The reaction conditions, including solvent, ratio, pH, and reaction time, were optimized systematically and the optimal one was reacted in dimethyl formamide at pH 8.2 with triethylamine at room temperature for 120 h. The in vitro stabilities in serum, simulated gastric juice, and intestinal fluid were tested, and improved dramatically compared with TxID. The PEG-modified peptide was functionally tested on α3β4 nicotinic acetylcholine receptor (nAChR) heterologously expressed in *Xenopus laevis* oocytes. The DSPE-PEG-TxID showed an obvious inhibition effect on α3β4 nAChR. All in all, the PEG modification of TxID was improved in stability, resistance to enzymatic degradation, and may prolong the half-life in vivo, which may pave the way for the future application in smoking cessation and drug rehabilitation, as well as small cell lung cancer.

## 1. Introduction

Nicotinic acetylcholine receptors (nAChRs)—a class of cell membrane proteins—are ubiquitous receptors in mammalian cells which play an important role in neuroscience, physiology, and clinical research. The nAChRs mediate learning, pain, exercise, memory, and other physiological functions by involving the release of a variety of neurotransmitters. Therefore, nAChRs are targets for the screening and treatment of many intractable diseases [1,2,3,4]. 

Conotoxins are active peptides derived from the venom of marine mollusks—cone snails—used for hunting and defense. There are around 700 species of cone snails in the world, and each snail can secrete thousands of different conopeptides. These conotoxins usually consist of 10–40 amino acids and are rich in cysteine. According to their cysteine framework and the conserved sequence of signal peptides, they are further divided into more than 20 superfamilies, such as A, M, P, O, S, T, and V. According to pharmacological targets, they are further divided into pharmacological families such as α, μ, ω, κ, δ, and γ [5,6,7]. Conotoxins are important sources of new drugs. They have attracted the attention of scientists from all over the world due to their great potential as drug leads [8,9].

Peptide drugs generally have disadvantages such as poor stability and short half-life [10]. In order to achieve a therapeutic effect, it is necessary to increase the dose or frequency of administration, and these actions not only increase the pain and economic burden of the patient, but also cause serious side-effect problems, which severely limits their wide application. Therefore, in order to improve the half-life of the peptide drug, various modifications are essential to increase its stability [11]. PEGylation modification is an effective method and technique for solving some of the problems. The activated polyethylene glycol is coupled to the polypeptide molecule, and the effect of prolonging the half-life is achieved by increasing the volume of the drug molecule, and the resistance to the enzymatic degradation effect is improved by masking the enzymatic cleavage site. It is one of the most effective long-acting protein and peptide preparation technologies recognized internationally [12,13]. Synthetic amphiphilic copolymers are ideal tools for drug delivery because they are highly versatile in terms of composition and architecture. Among the different polymers, 1,2-distearoyl-sn-glycero-3-phosphoethanolamine-N-[hydroxysuccinimidyl(polyethylene glycol)-2000] (DSPE-PEG-NHS) is currently one of the most commonly used modifiers attracting much attention due to its biomaterial characteristics, including safety, biocompatibility, and low toxicity [14,15,16]. Peptide modification by covalently bonded PEG can effectively overcome the short half-life and poor stability of peptide drugs and has achieved good results. A variety of PEG-modified protein peptides have been approved by the FDA for clinical treatment [17,18], as shown in Table 1.

**Figure 1:**
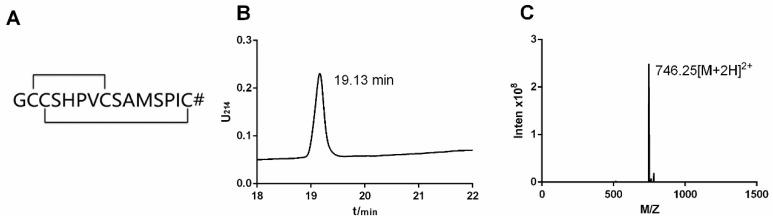
(**A**) Sequence and disulfide bond connection of TxID, # represents a C-terminal amide; (**B**) RP-UPLC chromatogram of TxID; (**C**) ESI-MS data of TxID.

α-Conotoxins are blockers acting specifically on different nAChR subtypes. α-Conotoxin TxID was cloned from Hainan native *Conus textile* in our laboratory, which holds the independent intellectual property rights. Its primary structure contains 15 amino acids and two disulfide bonds between cysteines I-III and II-IV, and the C-terminus is amidated. TxID belongs to the conotoxin α4/ 6 subfamily with a relative molecular mass of 1489.5 Da, as shown in Figure 1A [19,20]. α-Conotoxin TxID inhibits α3β4 nAChR subtype and it is a potential painkiller for the treatment of neuropathic pain. It is also a potential drug to treat addiction and small cell lung cancer [8,21,22]. 

However, like most peptides, α-conotoxin TxID also has the disadvantage of poor stability and a short half-life in biological systems, which will limit the clinical application. Herein, we synthesized the N-terminal modified TxID, DSPE-PEG-TxID, by a nucleophilic substitution reaction for the first time. The reaction conditions, including solvent, ratio, pH, and reaction time, were optimized systematically and the optimal one was reacted in dimethyl formamide at pH 8.2 with triethylamine at room temperature for 120 h. The in vitro stabilities in serum, simulated gastric juice, and intestinal fluid were tested and improved dramatically compared with TxID. The PEG-modified peptide was functionally tested on α3β4 nAChR heterologously expressed in *Xenopus laevis* oocytes. These studies will greatly improve the development of new drugs from TxID.

## 2. Results

### 2.1. Synthesis and Identification of DSPE-PEG-TxID

In this study, the standard Fmoc solid phase peptide synthesis strategy was used for the peptide chain. α-Conotoxin TxID, as shown in Figure 1A, was successfully synthesized by two-step oxidation and purified by preparative HPLC. The purity was monitored by RP-UPLC and the molecular weight was identified by ESI-MS, as shown in Figure 1B,C. The retention time of TxID was 19.13 min. The purity of all fully folded peptides was above 95%. As shown in Figure 1C, ESI-MS was used to confirm that the TxID has a molecular weight of 1489.00 Da with *m*/*z* of 746.25 Da [M + 2H]^2+^, which is consistent with its theoretical average mass of 1489.68 Da.

**Figure 2:**
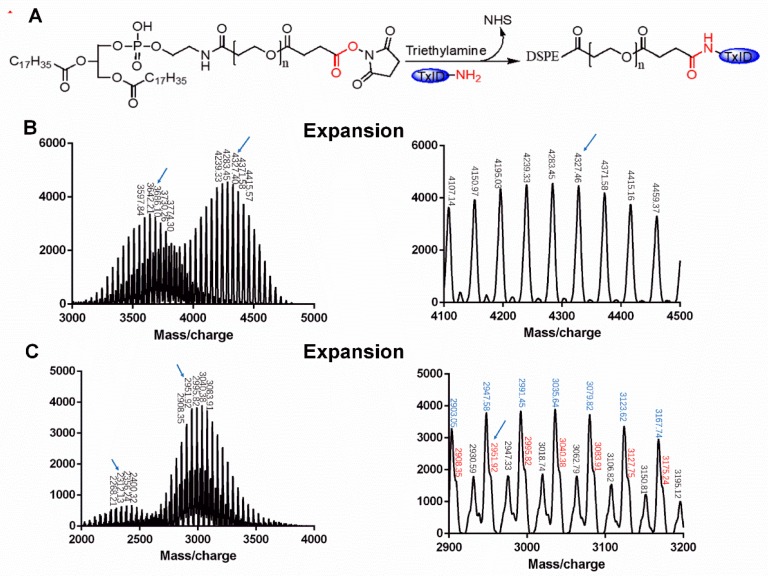
(**A**) Synthesis scheme of DSPE-PEG-TxID; (**B**) MADLI-TOF spectrum of DSPE-PEG-TxID; (**C**) MADLI-TOF spectrum of DSPE-PEG-NHS.

The targeting copolymer DSPE-PEG-TxID was synthesized by a nucleophilic substitution reaction between the NHS and the N-terminal of TxID, as shown in Figure 2A. According to the HPLC chromatogram, the unreacted TxID retention time was 19.13 min, as shown in Figure 1B, which means the peak of TxID was not disturbed by DSPE-PEG-NHS. As shown in the MALDI-TOF MS spectrum, the molecular weight (MW) of the final products are all consistent with the theoretical MW of DSPE-PEG-TxID. For example, two of the monomers with the MW of 3686.10 and 4327.40 Da, as shown in Figure 2B, are consistent with the theoretical MW of DSPE-PEG-TxID derived from the DSPE-PEG-NHS with MW of about 2312.13 and 2951.92 Da, as shown in Figure 2C, confirming that the obtained products were the target compound DSPE-PEG-TxID. In the locally enlarged Figure 2C, as sodium and potassium ions were inevitably mixed in the mass spectrometry detection process, the red peak represents the molecular weight of DSPE-PEG-NHS, the black peak represents the molecular weight of DSPE-PEG-NHS plus sodium ions, and the blue peak represents the molecular weight of DSPE-PEG-NHS plus potassium ions. 

**Figure 3:**
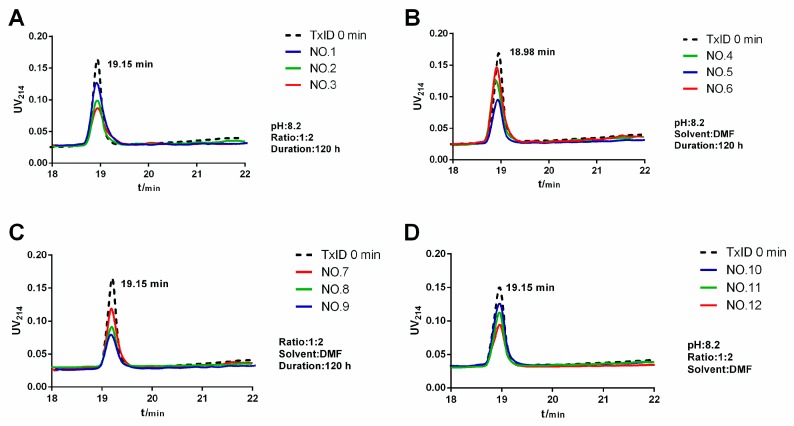
Chromatograms of reaction optimization under various conditions, the changes in solvent (**A**), ratio of polypeptide to PEG (**B**), pH (**C**) and reaction time (**D**) (see Table 2).

System optimization was monitored by HPLC to determine the remaining amount of the polypeptide under different reaction conditions, with the remaining minimum indicating that the polypeptide had the highest conversion under the reaction conditions and vice versa. The chromatograms of the optimization scheme under different conditions are shown in Figure 3.

The chromatograms of the optimized scheme are summarized in Table 2. When phosphate buffer saline (PBS) was used as a solvent, as shown in Table 2 (entry 1), the conversion was incomplete and the remaining amount was 63.2%. After replacing with DMSO or DMF, as shown in Table 2 (entry 2 and 3), the remaining amount was about 35%. Although the use of DMSO can decrease the remaining amount, as shown in Table 2 (entry 2), DMSO was difficult to remove in subsequent experiments, which was not conducive to the purification and separation of the reaction product. Then we examined the effect of different peptide/PEG molar ratios on the yield of the reaction, as shown in Table 2 (entry 4, 5, and 6); as the concentration of the polypeptide increases, the occurrence of side reactions increases. Reducing the concentration of the polypeptide facilitates the obtaining of a single modified product, which also helps to reduce costs. Weak alkaline conditions are beneficial to the reaction, as shown in Table 2 (entry 7, 8, and 9), and the random modification of PEG can be effectively reduced by changing the pH of the reaction system. Pleasantly, we found that a pH of 8.2 was a good choice, as shown in Table 2 (entry 9). The reaction time also had an influence on the modification, which was reflected in the conversion degree of the polypeptide. At this stage, we strived to use reaction time to increase production. In this study, a long-term slow and sufficient reaction strategy was selected, as shown in Table 2 (entry 12). 

### 2.2. Potency of TxID and DSPE-PEG-TxID on α3β4 nAChR

**Figure 4:**
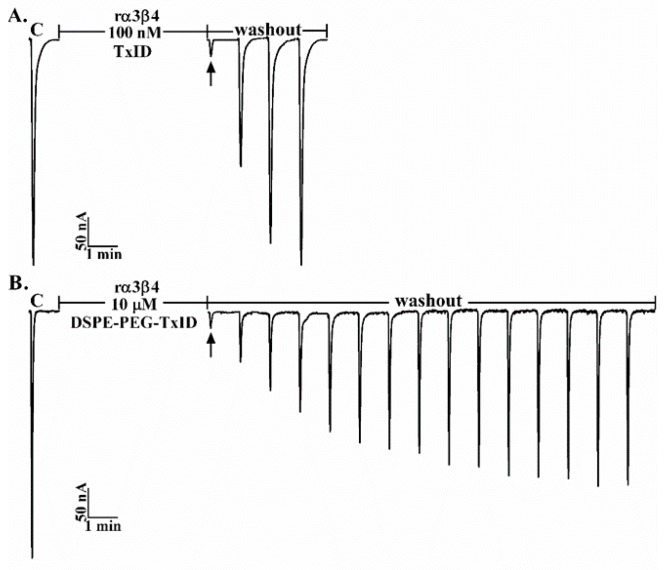
α-Conotoxin TxID and DSPE-PEG-TxID were tested on neuronal rat α3β4 nAChR subtype expressed in *Xenopus laevis* oocytes. The representative current traces showing the inhibition of rat α3β4 ACh-evoked currents by TxID (**A**) and DSPE-PEG-TxID (**B**).

α4/6-Conotoxin TxID is a polypeptide that potently blocks rat α3β4 nAChR with a unique selectivity profile [8]. We observed that greater than 90% blockade of rat α3β4 nAChR ACh-evoked currents was obtained with 100 nM TxID, as shown in Figure 4A. As also shown, TxID blockade of α3β4 nAChR was relatively quickly reversible, requiring only 3 min washout while the recovery of over 90% blockage was accomplished with 10 μM DSPE-PEG-TxID more than 10 min after DSPE-PEG-TxID washout, as shown in Figure 4B. Therefore, DSPE-PEG-TxID was still effective in inhibiting the α3β4 receptor, relative to TxID with slower reversible recovery.

### 2.3. Stability of TxID and DSPE-PEG-TxID

**Figure 5:**
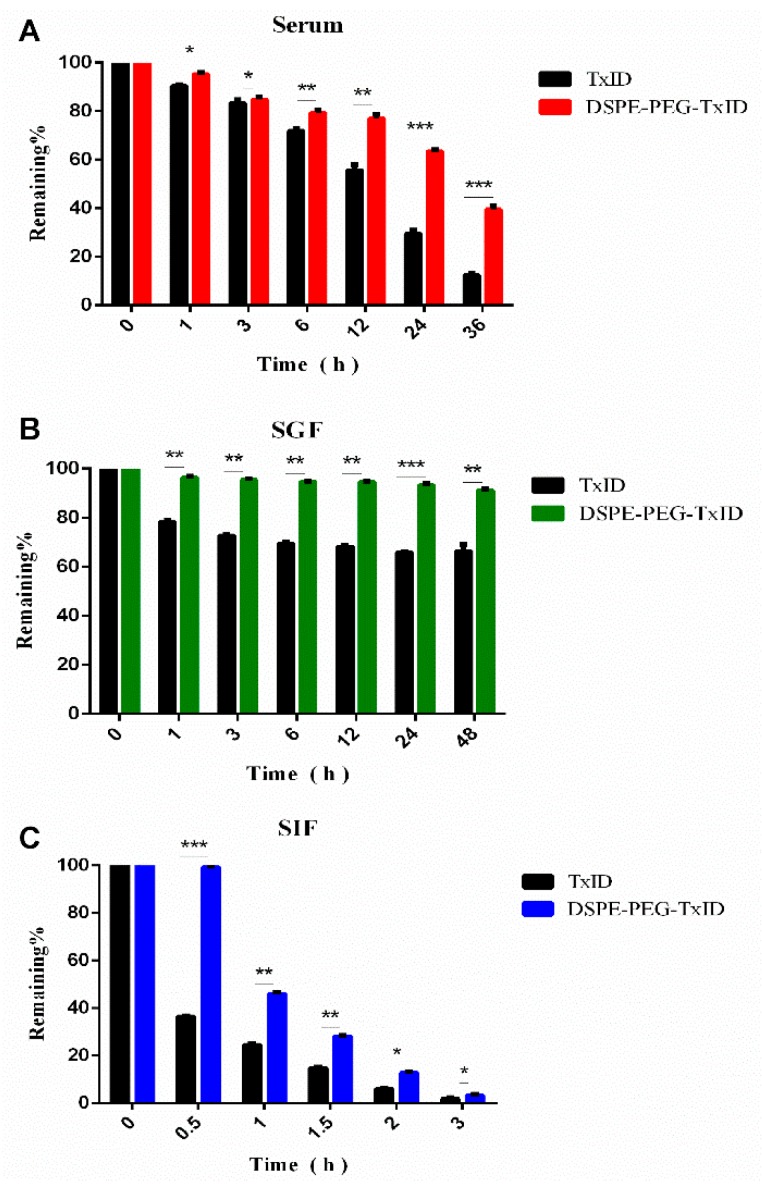
Stability of TxID and DSPE-PEG-TxID in serum (**A**), simulated gastric fluid (SGF) (**B**), and simulated intestinal fluid (SIF) (**C**). * *p* < 0.05, ** *p* < 0.01, *** *p* < 0.001, compared with the TxID.

The stability of TxID and DSPE-PEG-TxID were tested in human serum, as shown in Figure 5A. RP-HPLC was used to determine the degree of degradation of the peptide. After 3 hours’ incubation in human serum, there was little difference between TxID and DSPE-PEG-TxID. After 24 h, the unmodified polypeptide was reduced to <30%. After 36 h, it was reduced to 12%. The PEG-modified polypeptide was significantly stable in serum and remained >63% even after 24 h of incubation. Thus, the modified polypeptide is much more stable in human serum than the unmodified polypeptide, as shown in Figure 5A.

The main reason for limiting the spread of peptide drugs is that there is no pharmacological effect, and/or significant reduction in efficacy when administered orally, which is caused by poor stability of the polypeptide drug and absorption in the digestive system [23,24]. Enzymatic degradation was used to assess the stability of TxID in the gastrointestinal tract before and after PEG modification. TxID and DSPE-PEG-TxID were incubated with SGF (simulated gastric fluid) for 48 h at 37 °C, as shown in Figure 5B. The remaining amount of the unmodified polypeptide was observed to be 65.2 ± 2.0% after 48 h of incubation, while the remaining amount of the modified polypeptide was 92.1 ± 1.2%. The results indicated that the PEG-modified polypeptide was more stable within 48 h compared to the unmodified polypeptide.

Then, we incubated the two polypeptides separately in SIF (simulated intestinal fluid) before and after the modification at 37 °C, as shown in Figure 5C. The difference was significant after 0.5 h of incubation; the remaining amount of the TxID was 36.3 ± 2.0%, and the remaining amount of DSPE-PEG-TxID was 99.7 ± 2.0%. After 2 h, the remaining amount of the unmodified polypeptide was 5.8 ± 2.0%, and the remaining amount of the modified polypeptide was 13.1 ± 2.0%. Thus, the PEG-modified polypeptide is more stable within 2 h of SIF compared to the unmodified polypeptide.

## 3. Discussion

Due to structural characteristics, peptide drugs have many advantages, like good activity, specificity, and clear biological functions. They also have weaknesses, like a short half-life and unstable in vivo, which severely limits their wide application [25]. For example, the first conotoxin drug, MVIIA, was marketed in the United States on 28 December 2004 for analgesia in patients with refractory chronic pain, advanced cancer pain, and AIDS pain. However, MVIIA has low bioavailability and a short half-life, which reduces its drug compliance [26]. How to solve these key problems is an important direction that must be considered to effectively promote the development of the peptide drug industry. Polyethylene glycol chemical modification is an effective method and technology to solve the above problems and is also recognized as one of the most effective long-acting peptide formulation technologies.

The polyethylene glycol modification can effectively improve the stability, enhance the resistance to enzymatic degradation ability, and significantly reduce the immunogenicity of the drug. After half a century of development, a number of PEG-modified long-acting preparations have been successfully put into the market, and a considerable number of new modified products have entered the clinical arena, as shown in Table 1 [15,27].

It is certain that these bioactive molecules are chemically modified by polyethylene glycol, and the stability and resistance to enzymatic degradation ability are improved, which can effectively prolong the half-life and reduce the immunogenicity. These are similar to the results of this study. The stability and resistance to enzymatic degradation ability of TxID were improved by polyethylene glycol modification.

Biological activity of biopharmaceuticals requires physical stability, chemical stability, and the influence of different environmental factors, all of which should be assessed using appropriate techniques [28]. There are three isomers of TxID. The difference between them is that the disulfide bonds are connected in different ways. Among them, only the isomer with the I-III and II-IV disulfide pattern potently blocks rat α3β4 nicotinic acetylcholine receptors. Therefore, we used the Fmoc chemical synthesis method to synthesize TxID, which was identified by RP-UPLC and ESI-MS. According to previous reports, TxID has a similar IC_50_ for rat and human α3β4 nAChRs and has an analgesic effect on refractory pain in rats without drug addiction. Therefore, this was the first study to link TxID with DSPE-PEG-NHS. For polypeptide drugs, PEG modification can maximize the biological activity, improve the physical and chemical properties, and extend the half-life in vivo. The benefits of subsequent separation and purification should also be considered. Therefore, we conducted a preliminary study to optimize the PEG modification conditions from different solvents, reagents ratio, pH, and reaction time. The stability of peptides before and after modification was evaluated in human serum, simulated gastric juice, and simulated intestinal fluid, and to evaluate the stability of the peptide under various conditions before and after modification, including human serum and simulated gastrointestinal fluid. These results are important for PEG-modified polypeptides, which will help to determine the stability of the drug in vivo to ensure efficacy and reduce dosage.

In this study, the activity of the polypeptide was decreased after modification with PEG, and this result is consistent with the previous literature [29]. In general, the steric hindrance of the polypeptide changes due to the attachment of the PEG modifier, thereby affecting the activity. In this paper, the modification of the N-terminal amino group is simple and easy, the modified product is easy to control, and the activity loss is small [13]. In terms of stability, the stability of the modified TxID is higher than that of the unmodified one under different conditions, such as 100% serum, simulated gastric juice, simulated intestinal fluid, and the like. As the long chain of PEG interferes with the cleavage site and protects the polypeptide, which makes it more resistant under various stress conditions, the stability of TxID-PEG was increased. Taken together, our work extends our knowledge of TxID’s PEG modification and post-modification stability, providing sufficient data to further design TxID analogs with higher stability and affinity.

## 4. Materials and Methods 

N-hydroxysuccinimidyl-PEG2000-DSPE800 (NHS-PEG-DSPE, MW 2951.92) was purchased from Ponsure Biotechnology (Shanghai, China); α-conotoxin TxID (TxID, MW 1489.45) was synthesized by China Peptides Co. Ltd. (Shanghai, China); reversed-phase high-performance liquid chromatography (RP-HPLC) preparative C18 Vydac columns (10 μm, 22 × 250 mm) were obtained from Grace Vydac (Hesperia, CA, USA). Acetonitrile (ACN, HPLC grade) was purchased from Fisher Scientific (Pittsburg, PA, USA) and trifluoroacetic acid (TFA) was from Tedia Company (Fairfield, OH, USA). Human serum, pepsin, and trypsin were obtained from Sigma (St. Louis, MO, USA). All other solvents and reagents were of analytical grade and used as received.

### 4.1. Synthesis of DSPE-PEG-TxID 

In order to prepare highly pure active conotoxin TxID, a two-step oxidative synthesis using potassium ferricyanide and iodine was performed as previously described. For the linear peptides required for the two-step oxidation process, two kinds of protecting groups were used. Cys2 and Cys8 were protected by trityl (Trt); Cys3 and Cys15 were protected by acetylaminomethyl (Acm) [30,31]. In brief, cleavage of the peptide–resin complex was first performed using trifluoroacetic acid/phenol/water/TIPS (volume ratio 88:5:5:2). The crude peptide was then precipitated with cooled diethyl ether at 4 °C overnight, washed 3 times, and the granules were collected. The powder was freeze-dried and stored at −20 °C. A two-step oxidation protocol was used to perform the first disulfide bond by adding the peptide to an equal volume of a mixture of 20 mM potassium ferricyanide (K_3_[Fe(CN)_6_]) and 0.1 M Tris (pH 7.5) and at room temperature stirring for 45 min. The use of iodine oxidation removes the S-Acm protecting group and simultaneously forms a second disulfide bond, resulting the formation of globula TxID isomer. The crude TxID was purified by HPLC using a reversed-phase Vydac C18 column. The molecular weight of the peptide was determined by liquid chromatography mass spectrometry (UPLC: ACQUITY UPLC H-Class Bio; Mass spectrometry: Waters Xevo TQD, flow rate 1 mL/min, B solution containing 0.1% TFA in 90% acetonitrile solution, and solution A was ultrapure water containing 0.1% TFA). 

The design experimental steps were based on previous related literature references [32]. A copolymer of DSPE-PEG-TxID was synthesized by a nucleophilic substitution reaction between the NHS amino group and the N-terminal group of TxID. Briefly, TxID and DSPE-PEG-NHS in a molar ratio of 1:2 were dissolved in dry DMF and the pH of the mixture was adjusted to 8.2 with triethylamine. The reaction was carried out at room temperature for 120 h under moderate agitation. The coupling efficiency was determined by reversed-phase high-performance liquid chromatography (RP-HPLC) with a gradient elution and detection wavelength of 214 nm. The mobile phase was a mixture of Buffer A and Buffer B with a flow rate of 1.0 mL/min. Buffer A was deionized water containing 0.1% trifluoroacetic acid and Buffer B was acetonitrile containing 0.1% trifluoroacetic acid. A linear gradient of a 10–50% eluate B over 30 min. The resulting reaction mixture was then transferred to a dialysis bag (molecular weight cutoff = 2500 Da) and dialyzed against ultrapure water for 48 h to remove unreacted TxID. Finally, the dialysate was lyophilized and stored at −20 °C until use. The matrix-assisted laser desorption/ionization-time of flight (MALDI-TOF, the method is positive ion, linear, and the matrix is α-Cyano-4-hydroxycinnamic acid) mass spectrometer was used to identify the conjugates of TxID and DSPE-PEG-NHS.

### 4.2. Activity of DSPE-PEG-TxID on α3β4 nAChR

Capped cRNA for α3 or β4 subunit was acquired in vitro using a SP6 in vitro transcription kit (mMESSAGE mMACHINE; Ambion, Austin, TX) following plasmid linearization, cRNA of the various subunits was combined to give ~500 ng/µL of each subunit cRNA (α3 and β4 subunit were mixed at a molar ratio of 1:1). Xenopus oocytes were injected with an amount of ~50 nL of this mixture as described previously within 1 day of harvesting and incubated at 17 °C [33]. Oocytes were transferred to a 50 μL cylindrical oocyte recording chamber and perfused at a rate of ~2 mL/min with ND96 solution (96.0 mM NaCl, 2.0 mM KCl, 1.8 mM CaCl_2_, 1.0 mM MgCl_2_, 1 μM atropine, 5 mM HEPES, 0.1 mg/mL bovine serum albumin, pH 7.1–7.5) by a gravity-fed perfusion system. The membrane potential was clamped at −70 mV and the ACh-evoked currents were recorded with a two-electrode voltage-clamp amplifier Axoclamp 900B (Molecular Devices Corp., Sunnyvale, CA, USA). The oocyte was subjected once a minute to a 1 s pulse of 100 μM Ach, to measure the baseline responses; the oocytes were continuously perfused with ND96, during which nAChRs were automatically applied at a 5 min interval. nAChR-mediated currents were evoked by pipetting 100 μL of ACh-containing solutions into the bath with the perfusion stopped. Oocytes were preincubated with the peptide for ~5 min prior to nAChR application and subsequently nAChRs were co-applied together with the peptide. All recordings were made 2–4 days post injection and analyzed using Clampfit 10.2 software (Molecular Devices Corp., Sunnyvale, CA, USA) at room temperature (~22 °C). 

### 4.3. Stability Assessment of DSPE-PEG-TxID 

A 100% human serum was used to determine the stability of the peptide in serum before and after modification. The serum was centrifuged at 14,000× *g* for 10 min to remove lipids. It was then incubated at 37 °C for 15 min before the assay. The PEG-modified polypeptide and the unmodified polypeptide were incubated in human serum at a final concentration of 0.25 mM at 37 °C for 0, 1, 3, 6, 12, 24, and 48 h, respectively. Then, 30 μL of three replicate aliquots were taken at different periods. Each serum aliquot was quenched with 30 μL of 6 μm urea and incubated for 10 min at 4 °C. Then, each serum aliquot was treated with 30 μL of 20% trichloroacetic acid (TCA) at 4 °C for an additional 10 min to precipitate serum proteins. The precipitated serum protein was then centrifuged at 14,000× *g*. The supernatant of each sample was analyzed by RP-UPLC on an analytical column using a linear gradient of solvent B (0.1% TFA in 90% ACN) at a flow rate of 0.5 mL/min. The absorbance was monitored at 214 nm. Triplicate serum samples containing no peptide were also treated in the same manner and treated as controls at each time point. The remaining amount of the peptide was calculated based on the UPLC peak area change.

The stability of the polypeptide before and after modification by gastrointestinal enzymes was evaluated in simulated gastric fluid (SGF) and simulated intestinal fluid (SIF), respectively. For gastrointestinal stability studies, SGF and SIF were prepared according to the formula given in United States Pharmacopeia [34]. The polypeptides before and after the modification were respectively incubated in SGF for 48 h and in SIF for 180 min at 37 °C. SGF consisted of 9 g/L sodium chloride and 3 g/L pepsin from the porcine gastric mucosa and the pH was adjusted to 1.8 with hydrochloric acid. The SIF consisted of 9 g/L of sodium chloride that contained pancreatin and trypsin from bovine pancreas at 10 g/L each and 3 g/L of bile salts, and the pH was adjusted to 6.5 with sodium hydroxide. 

Investigations were made at 0, 1, 3, 6, 12, 24, and 48 h (SGF) and 0, 30, 60, 90, 120, and 180 min (SIF) incubations. The reaction mixture was analyzed by RP-UPLC and eluted with a linear gradient of 5%–40% solvent B at a flow rate of 0.5 mL/min, where solvent A was H_2_O + 0.1% TFA and solvent B was 90% ACN + 0.1% TFA. UV absorbance was detected at 214 nm.

## 5. Conclusions

In summary, current research focuses on PEG modification of α-conotoxin TxID and factors that affect the stability of both polypeptides before and after PEG modification. In this study, α-conotoxin TxID was synthesized from linear peptides by two-step oxidation and was purified and identified by HPLC and mass spectrometry. The conditions of PEG modification were optimized to obtain DSPE-PEG-TxID by nucleophilic substitution successfully. Biological activity and stability were measured simultaneously by TxID as a control. The results showed that DSPE-PEG-TXID still has inhibitory activity against α3β4 nAChR and its stability was improved in human serum, simulated gastric juice, and simulated intestinal fluid. In addition, this is the first reported stability analysis of TxID under different conditions before and after PEG modification. We anticipate that this study may substantially improve TxID structural modification, and further inform the design for drug preparation.

## Figures and Tables

**Table 1:** List of marketed PEG-modified drugs and indications.

Product	Company	Modifier	Indication	Time to Market
Rurioctocog Alfa Pegol	Schering-Plough	Growth Factor VIII	Hemophilia	2016
Pegloticase	Horizon Pharma plc	Recombinant Uricase	Gout	2016
Naloxegol	AstraZeneca	Opioid Receptor Agonist	Constipation	2015
Omontys	Affymax	PEG-Erythrocyte Stimulating Agent	Anemia	2012
Krystexxa	Savient	PEG-Porcine Uricase	Gout	2010
Cimzia	UCB	PEG-TNF Antibody Fab Segment	Rheumatoid Arthritis	2008
Mircera	Roche	PEG-EPO	Anemia	2007
Pegaptanib	NeXstar	VEGF Receptor Agonist	Senile Macular	2005
Macugen	OSI/Pfizer	PEG-Nucleic Acid Ligand	Senile Macular	2004
Pegvisomant	Pfizer	EG-Growth Hormone Antagonist	Limb Hypertrophy	2003

**Table 2:** Reaction optimization under various conditions. PBS: phosphate buffer saline.

Number	Solvent	Peptide: PEG	pH	Duration (h)	% Remaining Amount
1	PBS				63.28
2	DMSO	1:2	8.2	120	34.16
3	DMF				36.02
4		1:1			66.84
5	DMF	1:2	8.2	120	31.55
6		2:1			85.88
7			6.2		47.46
8	DMF	1:2	7.2	120	31.64
9			8.2		29.94
10				54	68.25
11	DMF	1:2	8.2	78	48.65
12				120	34.61

## Abbreviations

**Abbreviation**	**Full name in English**
CTx	Conotoxin
nAChR	Nicotinic Acetylcholine Receptor
TFA	Trifluoroacetic Acid
RP-HPLC	Reversed-Phase High-Performance Liquid Chromatography
DSPE-PEG-NHS	1,2-distearoyl-sn-glycero-3-phosphoeth-anolamine-N-[hydroxyl succinimidyl (polyethylene glycol)-2000]
DMF	N,N-Dimethylformamide
LC-MS	Liquid Chromatograph Mass Spectrometer
MADLI-TOF-MS	Matrix-Assisted Laser Desorption/ Ionization Time of Flight Mass Spectrometry
DMSO	Dimethyl Sulfoxide
PBS	Phosphate Buffer Saline
SGF	Simulated Gastric Fluid
SIF	Simulated Intestinal Fluid

## References

[1] Hurst R., Rollema H., Bertrand D. (2013). Nicotinic acetylcholine receptors: From basic science to therapeutics. Pharmacol. Ther..

[2] Albuquerque E.X., Pereira E.F.R., Manickavasagom A., Rogers S.W. (2009). Mammalian nicotinic acetylcholine receptors: from structure to function. Physiol. Rev..

[3] Gotti C., Clementi F. (2004). Neuronal nicotinic receptors: from structure to pathology. Prog. Neurobiol..

[4] Dong-Ting Z., Yong W.U., Zhu X.P., Luo S.L. (2016). Sensitivity of α-Conotoxin TxID on Stoichiometry of α3β4 Nicotinic Acetylcholine Receptors. Chin. Pharm. J..

[5] Luo S., Christensen S., Zhangsun D., Wu Y., Hu Y., Zhu X., Chhabra S., Norton R.S., McIntosh J.M. (2013). A novel inhibitor of α9α10 nicotinic acetylcholine receptors from *Conus vexillum* delineates a new conotoxin superfamily. PLoS ONE.

[6] Luo S., Zhangsun D., Harvey P.J., Kaas Q., Wu Y., Zhu X., Hu Y., Li X., Tsetlin V.I., Christensen S. (2015). Cloning, synthesis, and characterization of αO-conotoxin GeXIVA, a potent α9α10 nicotinic acetylcholine receptor antagonist. Proc. Natl. Acad. Sci. USA.

[7] Mannelli L.D.C., Cinci L., Micheli L., Zanardelli M., Pacini A., Mcintosh J.M., Ghelardini C. (2014). α-Conotoxin RgIA protects against the development of nerve injury-induced chronic pain and prevents both neuronal and glial derangement. Pain.

[8] Layla A.J., Michael M. (2009). α-Conotoxins as pharmacological probes of nicotinic acetylcholine receptors. Acta Pharmacol. Sin..

[9] Olivera B.M., Quik M., Vincler M., Mcintosh J.M. (2008). Subtype-selective conopeptides targeted to nicotinic receptors: Concerted discovery and biomedical applications. Channels.

[10] Tang L., Persky A.M., Hochhaus G., Meibohm B. (2004). Pharmacokinetic aspects of biotechnology products. J. Pharm. Sci..

[11] Sanchez-Ruiz J.M. (2010). Protein kinetic stability. J. Biophys. Chem..

[12] Pascal B., Chee-Youb W. (2009). PEG-modified biopharmaceuticals. Exp. Opin. Drug Deliv..

[13] Fishburn C.S. (2010). The pharmacology of PEGylation: Balancing PD with PK to generate novel therapeutics. J. Pharm. Sci..

[14] Tong S.W., Xiang B., Dong D.W., Qi X.R. (2012). Enhanced antitumor efficacy and decreased toxicity by self-associated docetaxel in phospholipid-based micelles. Int. J. Pharm..

[15] Wu H., Zhu L., Torchilin V.P. (2013). pH-sensitive poly(histidine)-PEG/DSPE-PEG co-polymer micelles for cytosolic drug delivery. Biomaterials.

[16] Pasut G., Veronese F.M. (2012). State of the art in PEGylation: The great versatility achieved after forty years of research. J. Control. Release.

[17] Zündorf I., Dingermann T. (2014). PEGylation―A well-proven strategy for the improvement of recombinant drugs. Pharmazie.

[18] Li-Qiang P., Hai-Bin W., Jun L., Ying-Chun X., Chen Z., Shu-Qing C. (2013). Site-specific PEGylation of a mutated-cysteine residue and its effect on tumor necrosis factor (TNF)-related apoptosis-inducing ligand (TRAIL). Biomaterials.

[19] Kompella S.N., Andrew H., Clark R.J., Frank M., Adams D.J. (2015). Alanine scan of α-conotoxin RegIIA reveals a selective α3β4 nicotinic acetylcholine receptor antagonist. J. Biol. Chem..

[20] Luo S., Zhangsun D., Zhu X., Wu Y., Hu Y., Christensen S., Harvey P.J., Akcan M., Craik D.J., Mcintosh J.M. (2013). Characterization of a Novel α-Conotoxin TxID from Conus textile that Potently Blocks rat α3β4 Nicotinic Acetylcholine Receptors. J. Med. Chem..

[21] Napier I.A., Klimis H., Rycroft B.K., Jin A.H., Alewood P.F., Motin L., Adams D.J., Christie M.J. (2012). Intrathecal α-conotoxins Vc1.1, AuIB and MII acting on distinct nicotinic receptor subtypes reverse signs of neuropathic pain. Neuropharmacology.

[22] Improgo M.R., Soll L.G., Tapper A.R., Gardner P.D. (2013). Nicotinic acetylcholine receptors mediate lung cancer growth. Front. Physiol..

[23] Robinson S.D., Norton R.S. (2014). Conotoxin gene superfamilies. Mar. Drugs.

[24] Wang J., Yadav V., Smart A.L., Tajiri S., Basit A.W. (2015). Toward oral delivery of biopharmaceuticals: An assessment of the gastrointestinal stability of 17 peptide drugs. Mol. Pharm..

[25] Maddux N.R., Joshi S.B., Volkin D.B., Ralston J.P., Middaugh C.R. (2011). Multidimensional methods for the formulation of biopharmaceuticals and vaccines. J. Pharm. Sci..

[26] Dai Q.Y., Liu F.Y., Zhou Y.R. (2003). The synthesis of SO-3, a conopeptide with high analgesic activity derived from *Conus striatus*. J. Nat. Prod..

[27] Clark R.J., Jensen J., Nevin S.T., Callaghan B.P., Adams D.J., Craik D.J. (2010). The Engineering of an Orally Active Conotoxin for the Treatment of Neuropathic Pain. Angew. Chem. Int. Ed. Engl..

[28] Mross K., Richly H., Fischer R., Scharr D., Büchert M., Stern A., Gille H., Audoly L.P., Scheulen M.E. (2013). First-in-human phase I study of PRS-050 (Angiocal), an Anticalin targeting and antagonizing VEGF-A, in patients with advanced solid tumors. PLoS ONE.

[29] Tamizi E., Jouyban A. (2016). Forced degradation studies of biopharmaceuticals: Selection of stress conditions. Eur. J. Pharm. Biopharm..

[30] Luo S., Zhangsun D., Schroeder C.I., Zhu X., Hu Y., Wu Y., Weltzin M.M., Eberhard S., Kaas Q., Craik D.J. (2014). A novel α4/7-conotoxin LvIA from *Conus lividus* that selectively blocks α3β2 vs. α6/α3β2β3 nicotinic acetylcholine receptors. FASEB J..

[31] Zhangsun D., Zhu X., Wu Y., Hu Y., Kaas Q., Craik D.J., Mclntosh J.M., Luo S. (2015). Key residues in the nicotinic acetylcholine receptor β2 subunit contribute to α-conotoxin LvIA binding. J. Biol. Chem..

[32] Mei D., Lin Z., Fu J., He B., Gao W., Ma L., Dai W., Zhang H., Wang X., Wang J. (2015). The use of α-conotoxin ImI to actualize the targeted delivery of paclitaxel micelles to α7 nAChR-overexpressing breast cancer. Biomaterials.

[33] Defu C., Wei G., Bing H., Wenbing D., Hua Z., Xueqing W., Jiancheng W., Xuan Z., Qiang Z. (2014). Hydrophobic penetrating peptide PFVYLI-modified stealth liposomes for doxorubicin delivery in breast cancer therapy. Biomaterials.

[34] Oba T., Matsunaka R., Dai N., Yamane K. (1974). The United States Pharmacopeia.

